# Plasmon-Assisted Trapping of Single Molecules in Nanogap

**DOI:** 10.3390/ma16083230

**Published:** 2023-04-19

**Authors:** Maoning Wang, Jieyi Zhang, Adila Adijiang, Xueyan Zhao, Min Tan, Xiaona Xu, Surong Zhang, Wei Zhang, Xinyue Zhang, Haoyu Wang, Dong Xiang

**Affiliations:** 1 Institute of Modern Optics and Center of Single-Molecule Science, Tianjin Key Laboratory of Micro-Scale Optical Information Science and Technology, Nankai University, Tianjin 300350, China; 2 School of Materials Science and Engineering, Smart Sensing Interdisciplinary Science Center, Nankai University, Tianjin 300350, China

**Keywords:** plasmonic optical trapping, single molecule study, molecular scale electronics, molecular junction

## Abstract

The manipulation of single molecules has attracted extensive attention because of their promising applications in chemical, biological, medical, and materials sciences. Optical trapping of single molecules at room temperature, a critical approach to manipulating the single molecule, still faces great challenges due to the Brownian motions of molecules, weak optical gradient forces of laser, and limited characterization approaches. Here, we put forward localized surface plasmon (LSP)-assisted trapping of single molecules by utilizing scanning tunneling microscope break junction (STM-BJ) techniques, which could provide adjustable plasmonic nanogap and characterize the formation of molecular junction due to plasmonic trapping. We find that the plasmon-assisted trapping of single molecules in the nanogap, revealed by the conductance measurement, strongly depends on the molecular length and the experimental environments, i.e., plasmon could obviously promote the trapping of longer alkane-based molecules but is almost incapable of acting on shorter molecules in solutions. In contrast, the plasmon-assisted trapping of molecules can be ignored when the molecules are self-assembled (SAM) on a substrate independent of the molecular length.

## 1. Introduction

Accurately controlling nanometer-scale objects has attracted huge interest due to the broad prospects in medical, biological, life, and chemical applications [1,2]. Since the invention of optical tweezers by Arthur Ashkin in 1970 [3], the capability to trap objects has been advanced from microns to hundreds of nanometers [4]. However, the updating of optical trapping technologies in smaller objects has encountered challenges due to the requirement of the focal spot size on the same order as focused light wavelength, which meets the restriction of light diffraction limitation [5]. Moreover, previous optical molecular trapping requires the employment of nanoparticles as a handle for functionalized molecules to bind on [1]. Harsh conditions, such as ultra-high vacuum (UHV) to reduce thermal fluctuations in laser cooling triatomic molecules, are required [6]. Furthermore, anti-Brownian electrokinetic (ABEL) trapping approaches require fluorescent molecules, and these molecules randomly move in an area of several hundreds of nanometers [7]. The above strategies in molecular trapping are novel in their own areas, but they inherently suffer limitations in direct trapping under less-demanding conditions towards much broader applications. Since the year 2000, plasmon-enhanced optical trapping (POT) has shrunk the object scale into tens of nanometers by confining an electromagnetic (EM) field into the nanoscale hotspots [8,9], but to date, this method still lacks effective trapping over single molecules smaller than 2 nm. The failure of direct trapping single molecules at fixed locations (e.g., nanogap) under room temperature has been mainly attributed to the lack of precisely controlled nanogap, such that localized optical force is not sufficient to prevail over Brownian motions in nanoscale volumes [10]. The second drawback of current POT investigations is the lack of approaches to simultaneously characterizing the movement of a sub-10 nm single molecule [11]. To overcome these major obstacles, three substantial aspects need to be considered. The first factor to consider is the novel construction of nanostructure to precisely adjust the nanogap and simultaneously obtain sufficient optical force [12,13]. The second factor to take into account is a method to be able to characterize the trapping events of a single molecule at a length of sub-2 nm [11]. The third factor is to develop a novel statistical analysis approach to verify LSP-assisted molecular trapping. Thus, the scientific question in evidencing direct single-molecule capture by previous confirmation of optical microscope imaging is converted into the solid observation of count increments of molecular trapping events through statistical histograms.

In recent years, the wide applications of mechanically controllable break junctions (MCBJs) and scanning tunneling microscopy break junctions (STM-BJs) have rapidly promoted the investigation of electron transport across single-molecule junctions [14,15,16]. MCBJ approaches not only provide adjustable nanogapped electrode pairs to form molecular junctions, revealing the properties of single molecules, but also enable the generation of an LSP-induced hot spot with the remarkable enhancement of the EM field [17,18]. The formation of molecular junctions and the change of electrode–molecule contact configurations could be characterized by the recorded conductance traces [19,20]. Correspondingly, statistical analysis by compiling thousands of conductance traces into a conductance histogram provides an approach to render probabilities of molecular junctions formation [21,22]. Pioneering research groups employing MCBJs have reported that optical trapping will result in the enhancement of molecular junction formation as high as 29% for 4,4″-1 diamine-1,1′:4′,1″-terphenyl (TBDA), judged by the conductance histograms [23]. They also found that trapping probabilities have wavelength dependencies, from ~30% to over 60%, when incident light was adjusted from 514 nm to 691 nm [24]. Moreover, nearfield trapping by STM-BJs has been proven to increase the lifetime and robustness of molecular junctions by one order of magnitude [25]. The extension of junction lifetime will also lead to the increments of conductance histogram counts. Despite these significant achievements, one basic question still remains: could the plasmon in the nanogap truly trap nanometer-dimension single molecules, i.e., does the change of conductance histogram merely originate from the plasmon-assisted molecular trapping, rather than the enhancement of junction lifetime?

Herein, we carried out a systematic study to address the LSP-assisted trapping of single molecules by employing STM-BJ approaches and make the following clear observations: (1) The conductance histogram shows negligible changes upon plasmon excitation for the molecules self-assembled on the electrode surface. In contrast, the conductance histogram presents considerable changes upon plasmon excitation when the measurement is performed in solution with suspending molecules; (2) Only for those molecules with a longer length does the conductance histogram show obvious change upon plasmon excitation; (3) The length of the plateaus presented in the 2-dimension conductance traces almost remains unchanged upon plasmon excitation, which reflects the almost unchanged junction lifetimes. Based on these solid observations, we claim that localized plasmons truly can be used to trap the molecules inside the nanogap and thus promote the formation of molecular junctions in solutions, which contrasts the previous expectation that (sub) nanoscale molecules cannot be trapped because the applied optical force decreases exponentially as the acting object volume decreases.

## 2. Results and Discussion

**Figure 1:**
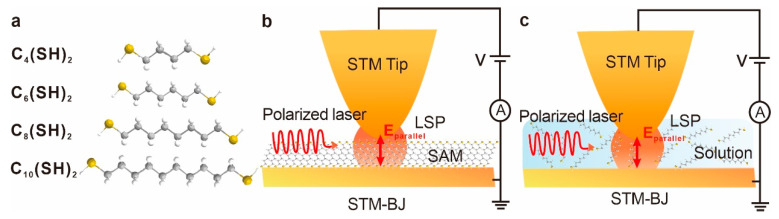
Schematics of STM-BJ measurements of probe molecules in SAM and solutions. (**a**) The chemical structures of four probe molecules are termed C_4_(SH)_2_, C_6_(SH)_2_, C_8_(SH)_2_, and C_10_(SH)_2_. (**b**) Schematic of STM-BJ measurements of probe molecules in SAM upon the illumination of polarized laser. The electric vector of polarized light is parallel to the electrode axis, denoted as E_parallel_. (**c**) Schematic of STM-BJ measurements of probe molecules in solution with 632 nm polarized laser.

As shown in Figure 1a, 1,4-butanedithiol (donated as C_4_(SH)_2_), 1,6-Hexanedithiol (C_6_(SH)_2_), 1,8-Octanedithiol (C_8_(SH)_2_), and 1,10-Decanedithiol (C_10_(SH)_2_) were employed as probe molecules. The molecular lengths for C_4_(SH)_2_, C_6_(SH)_2_, C_8_(SH)_2_, and C_10_(SH)_2_ are, respectively, 0.88 nm, 1.13 nm, 1.38 nm, and 1.63 nm, entering the sub-2 nm region. All these alkyl-based molecules have an anchoring group at two ends, which offers probabilities to form electrode–molecule–electrode junctions between two nanogapped electrodes. For the conductance measurement of SAMs-based junctions, the above four molecules were first diluted in acetonitrile solvents (1 mM). Subsequently, gold substrates were placed into the target solution for 24 h self-assembly, respectively. Finally, the substrates were blown dry with nitrogen for STM-BJ measurements. In contrast, we diluted these four molecules into 1,2,4-Trichlorobenzene (TCB) solvent (0.1 mM) for the STM measurements in solution because TCB is a stable nonpolar solvent that offers a wide access window for conductance measurement and evaporates at a slow speed [26].

Figure 1b,c shows the schematic of the STM-BJ experiments on SAMs and solutions, respectively. By employing a fiber laser (30 mW), a linearly polarized incident light (632 nm, ~10^6^ W/m^2^) was introduced into the STM-BJ setup, illuminating the nanogap with the electric vector parallel to the gold nanoelectrode axis to excite the localized plasmon [18,24,27]. To ensure that the laser spot was correctly focused on the nanoelectrodes, we carefully adjusted the laser outlet position to observe the clear overlapping of two neighboring laser spots across the interelectrode gap (one is on the end of the STM tip while the other in proximity is the reflection of the bottom gold film mirror).

**Figure 2:**
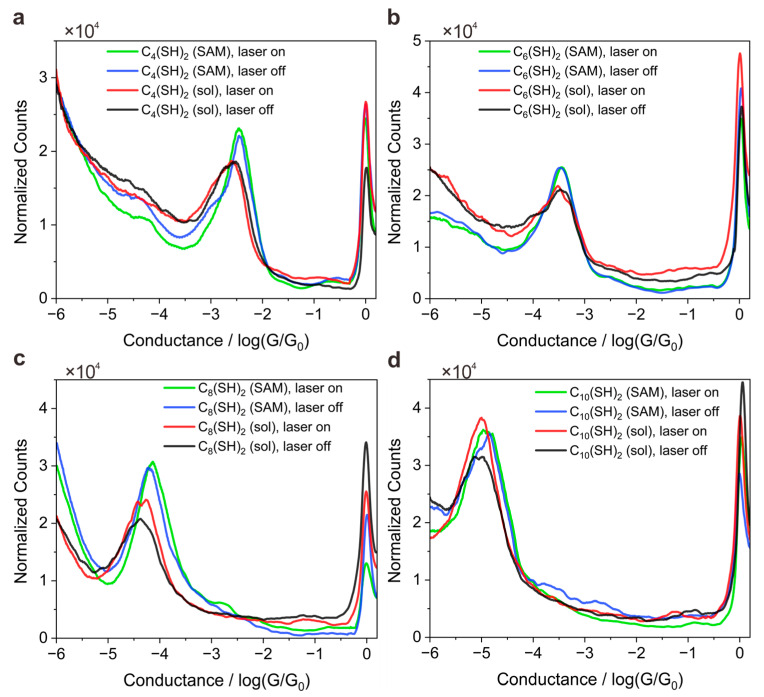
1D conductance histograms for C_n_(SH)_2_-based single-molecule junctions that comprise even n numbers. (**a**) 1D conductance histograms of C_4_(SH)_2_ with laser switching on or off in SAMs or in solutions. (**b**) 1D conductance histograms of C_6_(SH)_2_ with laser switching on or off in SAMs or in solutions. (**c**) 1D conductance histograms of C_8_(SH)_2_ with laser switching on or off in SAMs or in solutions. (**d**) 1D conductance histograms of C_10_(SH)_2_ with laser switching on or off in SAMs forms or in solutions. *G* is the conductance, equal to current/voltage. Conductance quantum *G*_0_ = $\frac{2e^{2}}{h}=77.3\;\mu S$, where *e* is the electron charge and *h* is Planck’s constant.

The STM-BJ experiments were performed at an applied bias of 100 mV. The current through the STM tips was measured at room temperature using a lab-built current-voltage converter with a sensitivity of 1 pA and a sampling rate of 30 kHz. Thousands of conductance traces were recorded and compiled into a 1-dimension (1D) histogram for each kind of molecule by repeatedly forming and then breaking point contacts between a gold STM tip and substrate covered with the SAMs or immersed in solutions. When two electrodes were bridged by a molecule via anchoring groups at two ends, a discernible molecular conductance plateau can thus be observed in a single recorded conductance–distance trace, which can be utilized to distinguish the formation of molecule junctions [28,29]. When the molecular junction breaks, the conductance trace continues to drop into quantum tunneling regions. As such, the positions of the pronounced conductance peaks as shown in Figure 2 indicate the most probable conductance values of the molecular junctions, while the counts in the y axis in Figure 2 show the counts of the measured data points of every specific bin size of conductance value across the ranges of 10^−6^ to 10^1^ *G*_0_.When more molecules are trapped into the nanogap through optical field gradient force, the probability of molecular junction formation will be obviously enhanced. Thus, the relative heights of the normalized histogram peaks could directly reflect yield contrasts of molecule junctions and thus indicate how much the plasmon trapping effects would lead to the changes of molecular junction yields [24].

Figure 2a–d shows the normalized conductance histogram of four probed molecules in SAMs or solutions, respectively. We note that the peak position in the normalized conductance histogram remains constantly independent of the light illumination for all four types of molecules, revealing that the light illumination does not change single-molecule conductance values. Meanwhile, we noted that peak height also does not obviously change for SAM junctions independent of the light illumination, indicating that light illumination does not change the yield of molecular junctions. With a more detailed examination, the yield of molecular junctions without illumination for C_4_(SH)_2_, C_6_(SH)_2_, C_8_(SH)_2_, and C_10_(SH)_2_ are approximately 24.3%, 22.1%, 38.6%, and 39.9%, respectively. Upon laser illumination, the yield of molecule junction changes to 22.4%, 21.8%, 37.0%, and 40.3%. Here, a molecular junction formation judged by the conductance trace is recognized as the appearance of 15 subsequent data points detected in the corresponding molecular conductance range. The change of junction yield at the level of 2% can be regarded as free fluctuations due to a minor deviation of STM tips, which means that the laser illumination and the resulting plasmonic force have minor effects on the yields of molecule junction for all four types of SAM junctions. This observation is reasonable because the SAM molecules bond to the bottom gold substrate, hindering the free movement of molecules, i.e., the optical gradient force smaller than the anchoring bond force is insufficient to trap/manipulate molecules [24,30].

In contrast to the SAM environment, the peak height increases upon light illumination, especially for the longer molecules (C_8_(SH)_2_ and C_10_(SH)_2_) as the experiment is performed in solutions. Upon light illumination, the molecular junction yield for C_4_(SH)_2_, C_6_(SH)_2_, C_8_(SH)_2_, and C_10_(SH)_2_ increases from 23.3%, 22.0%, 25.5%, and 32.6% to 24.7%, 24.5%, 33.7%, and 47.1%, respectively, which means the increments for longer C_8_(SH)_2_ and C_10_(SH)_2_ are 8.2% and 14.5% respectively. We attributed the molecular length dependent observation to LSP-assisted molecule trapping because of the plasmonic gradient force applying on the object scales with the third power of the object size so that the laser trapping effect might become much weaker as the molecule size decreases [31,32].

**Figure 3:**
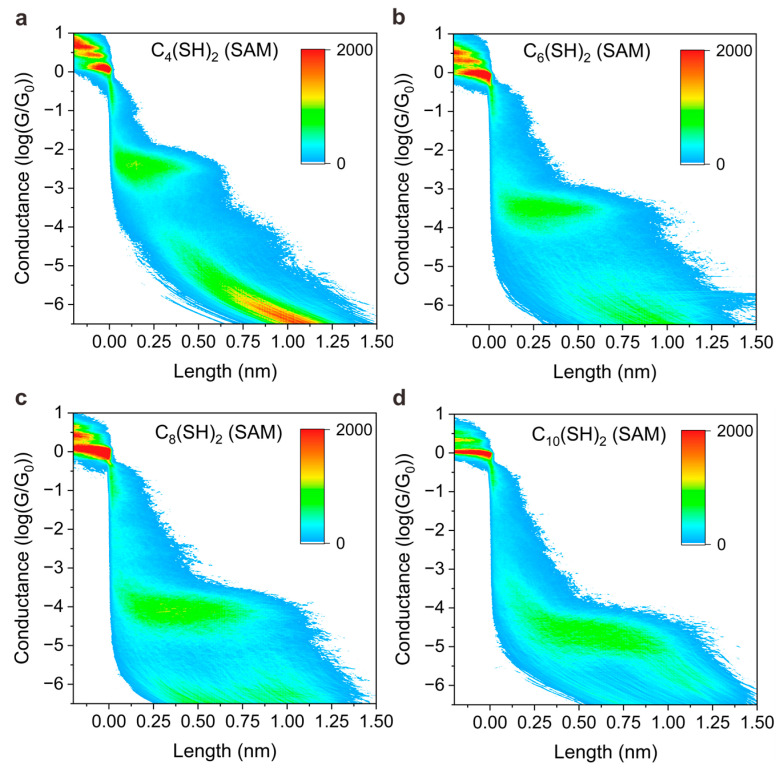
Experimental results of single-molecule conductance measurements with SAM-based junction. The 2D conductance–distance histograms for C_4_(SH)_2_ (**a**), C_6_(SH)_2_ (**b**), C_8_(SH)_2_ (**c**), and C_10_(SH)_2_ (**d**) were measured without laser illumination.

**Figure 4:**
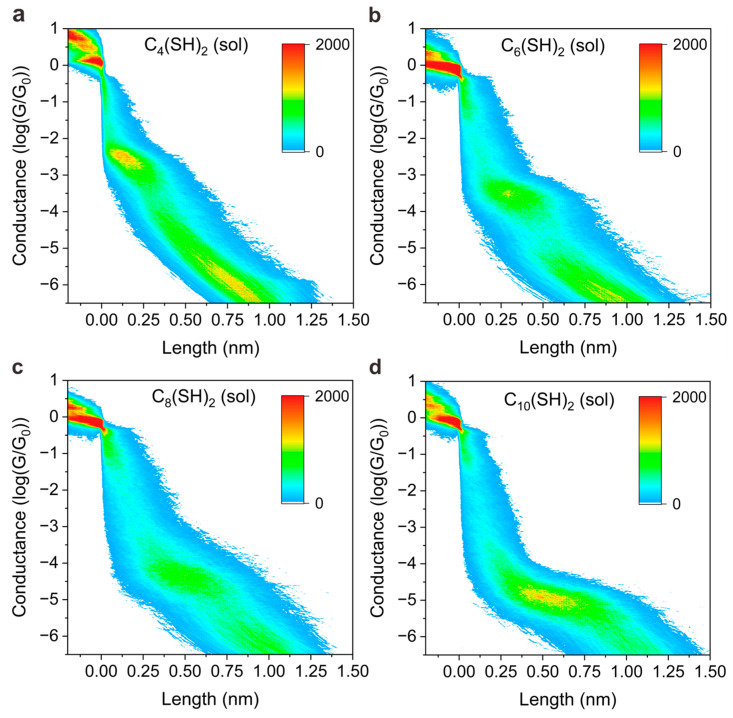
Experimental results of single-molecule conductance measurements in solutions. The 2D conductance–distance histograms for C_4_(SH)_2_ (**a**), C_6_(SH)_2_ (**b**), C_8_(SH)_2_ (**c**), and C_10_(SH)_2_ (**d**) were measured without laser illumination.

Furthermore, we noted that the peak position shifts to a lower value when the experimental environment is changed from solid SAMs to the solution, indicating the conductance of molecular junctions depends on the environment. To further dig into the conductance difference of junctions in SAM form and solution, and reveal the underlying mechanism, we plotted 2D colormaps (histogram) of conductance versus displacement distance of STM tip in different environments; see Figure 3 and Figure 4, respectively. The vertical displacement distance of STM tip is denoted as “length” in the x axis of Figure 3 and Figure 4. The 2D conductance–length histograms exhibit clear conductance plateaus, which correspond to the peak position as presented in 1D conductance. With close examination of 2D conductance histograms, we find that the conductance plateau shows a horizontal feature for SAMs-based junction without light illumination, as presented in Figure 3, whereas the conductance plateau shows a sloped behavior when the measurements were performed in solutions, as presented in Figure 4. The features of horizons or slopes represent how the junction conductance varies during the separation of tip and substrate.

The horizontal shape of the plateau indicates the junction conductance does not vary significantly during the junction elongation process, and the sloped shape of the plateau indicates the junction conductance varies across a range until a final break. The distinguished features presented in Figure 3 and Figure 4 can be interpreted by the different molecular adsorption mechanisms. The researchers have reported that physically absorbed bonding prevails in the SAM junctions for those molecules with thiol anchoring groups, while covalently, chemical adsorption dominates the bonding mechanism for the molecules in solution [33,34]. For SAMs, the molecules are normally in well-defined configuration so that physically absorbed species bind predominantly to undercoordinated gold adatoms [35], whereas chemically absorbed thiols out of order in solutions can adopt multiple contact geometries, exhibiting different anchoring configurations during elongation [36,37].

**Figure 5:**
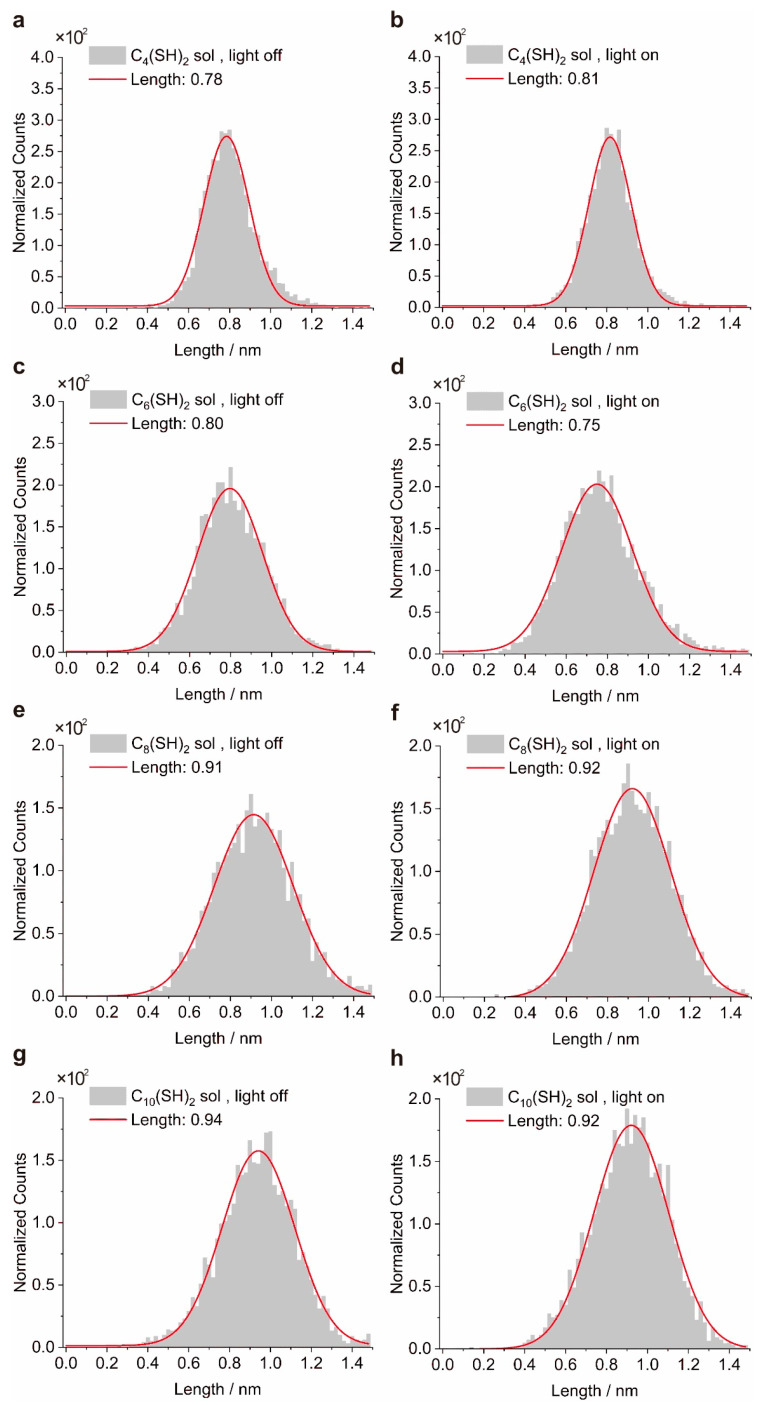
The Gaussian distributions of molecular conductance plateau lengths with light on or off. The data are extracted from 2D conductance histograms in Figure 4. The left column represents plateau length distributions for C_4_(SH)_2_ (**a**), C_6_(SH)_2_ (**c**), C_8_(SH)_2_ (**e**), and C_10_(SH)_2_ (**g**) solutions under dark conditions. The right column correspondingly represents plateau length distributions for C_4_(SH)_2_ (**b**), C_6_(SH)_2_ (**d**), C_8_(SH)_2_ (**f**), and C_10_(SH)_2_ (**h**) solutions at 632 nm illumination.

It is known that the lifetime of molecular junctions can also strongly affect the peak height presented in the 1D conductance histogram, e.g., a longer lifetime of molecule junction even with fixed junction yield can still generate a more pronounced peak [38,39]. To exclude the possibility that the changes in peak height originate from the change in the junction’s lifetime rather plasmon-assisted trapping, we extracted plateau length distributions of the junctions under dark conditions or light illumination as well (Figure 5). The lifetime of the junction is proportional to the plateau length of the junction, and the estimation of lifetime can be calculated by multiplying the number of data points recorded in the plateau range and the sampling rates of the measurement system. After Gaussian fitting, we found that the most probable plateau lengths in the distributions do not increase obviously upon light illumination. This observation indicates that the lifetime of the molecular junctions remains unchanged upon light illumination and thus the changes in peak height presented in the histogram truly originate from the changes in the junction yield.

## 3. Conclusions

In our work, STM-BJ renders a feasible approach to simultaneously achieve plasmonic molecular capturing and in situ single-molecule conductance sensing with size down to sub-2 nm in solution. This novel indicator of junction formation probabilities to characterize trapping capability is beyond the traditional limitations of highly-focused laser and the narrow selection of target materials. We demonstrate the nearfield trapping ability of both SAMs and molecular solutions for four probe molecules by quantifying the relative height of normalized counts of molecular junctions. For SAM, non-obvious changes in normalized counts have been observed, possibly because of the insusceptible immobilizing properties. Meanwhile, for longer C_8_(SH)_2_ and C_10_(SH)_2_ molecules in solutions, the considerable increments of junction yield demonstrate the plasmonic trapping effects occurring in flexible solution environments. Most importantly, we prove that the change of peak heights in the 1D conductance histogram does not originate from the change of the junction’s lifetime (judged by the length of conductance plateau), thus confirming that the increase of conductance peak truly originates from the plasmon-assisted trapping. Thus, we present an effective plasmon-assisted enhancement of molecular junction formation probabilities by a non-invasive combination of optical trapping and STM-BJ platform. Our work advances the understanding of single-molecule optical trapping and provides wide applicability to diverse sets of target molecules and electrode materials, thus paving the way for improved single-molecule sensing, manipulation, and recognition in chemical, biological, medical, and materials sciences.

## 4. Materials and Methods

Sample preparation. The bottom gold substrate was magnetron-sputtered by 2 nm Cr and 200 nm gold on silicon. The STM tip was prepared by burning one end of a gold wire (99.999%, 0.25 mm in diameter) with a methane flame. For measurement with SAM, the substrates were thoroughly rinsed with ethanol and blown dry with nitrogen after the self-assembling process.

Optical setup. We utilized a fiber laser (632 nm, Beijing Laserwave OptoElectronics Technology Co., Ltd., Beijing, China) to illuminate the junctions. The laser intensity was 30 mW by choosing 100 mA electrical current on laser control panel. A linear polarizer with vertical polarization was placed in the middle of laser source and STM break junctions.

Conductance measurement. The single-molecule conductance measurements of target molecules were performed using the STM-BJ technique. A positive 100 mV bias voltage was applied to the STM tip and the bottom electrode was connected to the ground. A feedback system was utilized to control the piezo for the STM tip, repeatedly forming and then breaking point contacts between a gold STM tip and substrate. The conductance trace was recorded as a function of tip–substrate displacement (denoted as “length” in the main text). These conductance–length traces exhibit steps at integer multiples of the conductance quantum *G*_0_ and additional steps appear at a lower conductance, marking that molecules bridge two electrodes to form molecular junctions. The conductance traces recorded during the electrode separation process were used to construct the 1D and 2D conductance histograms.

Generation of conductance histogram. Notably, the tip may be gradually distorted after long-time measurements, which may result in systematical error. To avoid this problem, we alternatively switched on or off the laser every five minutes and classified corresponding data into two packages according to dark or illuminating conditions.

Data processing. The total counts (y axis) before normalization in Figure 2 are data points located at different conductance ranges in 1D histogram, statistically collected by data acquisition units. Approximately 3600–3800 conductance traces were compiled into each conductance histogram. We set the 1D histogram under dark as a baseline and normalized the corresponding 1D histogram under light by dividing the respective ratio of conductance trace counts for meaningful histogram comparison. For the example of C_4_(SH)_2_ solutions under illumination and dark, the ratio of conductance trace counts is 3728/3676 = 1.014. For all the probe molecules of C_4_(SH)_2_, C_6_(SH)_2_, C_8_(SH)_2_, and C_10_(SH)_2_, the normalization method is identical unless otherwise noted. As for the calculation of molecular junction yields, we recognized a conductance trace as successful formation of molecular junction by the appearance of at least 15 subsequent data points detected in the corresponding molecular conductance range. Finally, the numbers of these molecular junction traces are counted and then divided by total counts of junction traces to obtain molecular junction yields. Total counts of junctions include both tunneling junctions without the trap of molecules and molecular junctions.

## Data Availability

Not applicable.
